# Catatonia Development in a Patient with Bipolar Disorder Following Electroconvulsive Therapy: A Case Report

**Published:** 2019-01

**Authors:** Azadeh Mashayekhi, Alireza Ghayoumi

**Affiliations:** 1 Psychosomatic Medicine Research Center, Tehran University of Medical Sciences, Tehran, Iran.; 2 Department of Iranian Traditional Medicine, Tehran University of Medical Sciences, Tehran, Iran.

**Keywords:** *Bipolar Disorder*, *Catatonia*, *Electroconvulsive Therapy*, *Vitamin B12 Deficiency*

## Abstract

**Objective:** Electroconvulsive therapy (ECT) is a major treatment of catatonia; and catatonia development during electroconvulsive therapy is a highly surprising phenomenon. We present a patient with bipolar disorder who developed catatonia during ECT.

**Case Reports** **:** A 21-year-old woman, with a known case of bipolar disorder in manic phase without psychotic feature, history of long acting antipsychotic use, and severe B12 deficiency, was treated with ECT. Full catatonia syndrome developed after the fifth session of ECT.

**Conclusion: **In rare cases, catatonia can develop during ECT course in the presence of some precipitating factors. Thus, these precipitating factors should be eliminated as much as possible before the start of ECT course to prevent catatonia development.

According to Diagnostic and Statistical Manual of Mental Disorders, Fifth Edition (DSM-5), catatonia is defined as the presence of three or more of the following: catalepsy, waxy flexibility and stupor, agitation not influenced by external stimuli, mutism, negativism, posturing, mannerisms, stereotypies, grimacing, echolalia, and echopraxia (1).

Catatonia is believed to be caused by irregularities in the dopamine, gamma-aminobutyric acid (GABA), and glutamate neurotransmitter systems. It is often accompanied by an underlying neurological, psychiatric, or physical illness (2). Some developmental disorders, such as intellectual disability and autistic disorder, can put the patient at the risk of catatonia (3). Catatonia has also been linked to benzodiazepine and alcohol withdrawal (4, 5 and 6). Differential diagnosis of catatonia includes many conditions, such as non-catatonic stupor, encephalopathy, stroke, Parkinson’s disease, malignant hyperthermia, status epilepticus, autistic disorder, severe obsessive-compulsive disorder, and selective mutism.

The major treatments of catatonic state are benzodiazepines (lorazepam in particular) and ECT. 

In the refractory or life-threatening cases of catatonia, such as malignant catatonia or neuroleptic malignant syndrome, in which prolonged food refusal, specific posturing, or autonomic dysfunction can put the patients in dangerous condition, ECT is the choice of treatment (7). ECT can subside catatonia by modulation of neurotransmitters and some changes in brain perfusion in different sites (2).

Hence, onset of catatonia during the course of ECT is a paradoxical and much surprising phenomenon.

In this manuscript, described a patient with bipolar disorder who developed catatonic state during ECT. Most of cases that have been presented so far developed catatonia in the context of withdrawal from benzodiazepines. The presented patient had no history of benzodiazepine use but had severe vitamin B12 deficiency which could have a possible role in developing catatonic state.

## Case Report

A 21-year-old woman was referred with aggression, irritability, talkativeness, decreased need to sleep, and racing thoughts without psychotic features.

Her symptoms commenced about 3.5 years ago. She had two prior psychiatric hospitalizations 1.5 and three years ago. The first episode was depression and she had history of two suicidal attempts in the depressed phase. In the second episode, she experienced mania with psychotic features. Because of drug noncompliance after the second episode, a long acting antipsychotic agent (Flupentixol Decanoate) was prescribed for her once monthly. The latest injection was 1.5 months before admission, but she stopped taking Biperiden (4mg/day) and lithium carbonate (900mg/day) since five months ago.

 She had a family history of major depressive disorder in her mother and elder sister. She had not any medical comorbidities or history of brain trauma or autistic disorder. Moreover, she had no history of benzodiazepine or alcohol withdrawal.

Laboratory data at the admission time were within normal range except for vitamin B12 level which was in the range of severe deficiency (as the result of vegetarianism). The brain neuroimaging (MRI) revealed no abnormality. 

At admission, she had non-cooperative attitude, irritable mood with congruent affect, and pressure of speech without any hallucinations or delusions. She was well-oriented. Her vital signs were within normal levels. In medical examination, she had no tremor, rigidity, or any other extra pyramidal signs.

Drug regimen included lithium carbonate up to 1200 mg/day with 1 mg/dL of serum level, Quetiapine up to 600 mg/day, and Biperiden up to 4 mg/day. Within 3 weeks of non-responsiveness, lithium carbonate was tapered and discontinued within a week. The rest of the medications continued. Then, bitemporal ECT was given with energy level of 25% every other day. Duration of seizures was between 22 to 45 seconds. Anesthesia was induced by Atropine 0.5 mg IV, Succynil choline 25 mg IV, and Thiopental sodium 150 mg IV. The reason for the ECT was non-responsive to the medications, and the patient's family insisted for treatment as soon as possible due to fear of possible consequences of mania for the patient.

She developed some negativism after the fourth session of ECT, and after the fifth session, she developed full catatonic state including waxy flexibility, posturing, negativism, mutism, and echolalia.

Her vital signs were normal and she had not any autonomic dysfunction and extrapyramidal signs. The patient had no history of catatonia or neuroleptic malignant syndrome.

ECT and other psychotropic drugs were discontinued as soon as possible, and lorazepam was prescribed and increased up to 4mg/day twice a day.

The fulminant catatonia resolved after 4 days of treatment, and lorazepam was tapered after remission. Because of non-responsiveness to other antipsychotic agents, clozapine was started and its dose was escalated to 200 mg/day during 25 days until the patient’s discharge. After discharge, there was no sign of recurrence of catatonia in the follow- up sessions.

Informed consent was obtained from the patient for presentation.

## Discussion

In the presented case, a patient with bipolar disorder and most recent episode of mania but without psychotic feature had developed catatonia during the course of electroconvulsive therapy. She had no history of brain trauma or any other medical conditions. Also, she did not have any history of autistic disorder or benzodiazepine withdrawal. However, laboratory data demonstrated severe vitamin B12 deficiency. She had history of long acting antipsychotic agent use (flupentixol decanoate).

There are some reports on the emergence of catatonia during ECT in the literature. In 2004, Malur and Francis reported 4 cases of catatonia development during ECT. Four inpatients with affective disorders (3without prior catatonia) developed catatonia during a course of ECT. All 4 patients had been taking benzodiazepines, which were stopped 5–15 days before ECT (8). To date, 3 cases of emergence of catatonia during ECT had been reported in the absence of benzodiazepine withdrawal. In 1988, for the first time, Pandey and Sharma reported an ECT- induced catatonia in a 26- year- old woman with bipolar disorder in depressed phase (9).

In 2010, Shams et al. reported a schizoaffective disorder patient with a positive history of traumatic brain injury and no consumption of benzodiazepines, who became catatonic during ECT (10).

In 2013, Praharaj et al. reported a 31-year-old man with paranoid schizophrenia who developed catatonia during ECT without history of benzodiazepine withdrawal or any other medical precipitating factors (11).

The first probable etiology in our case was increased concentration of a long acting antipsychotic in central nervous system (CNS). Blood brain barrier permeability increases following ECT (12). The abovementioned increased permeability might have resulted in an increased antipsychotic concentration in CNS. This phenomenon may have caused induction of antipsychotic side effects, such as catatonia.

ECT changes the perfusion of several areas of the brain. Escobar et al. reported increased brain perfusion in the parietal, temporal, and occipital areas in catatonic patients with mood disorder during ECT treatment; however, no changes occurred in the brain perfusion of schizophrenic patients (12). On the other hand, Galynker et al. reported increased brain perfusion in the left parietal and motor area in a patient with schizoaffective disorder with catatonia during the course of ECT (13). These changes in brain perfusion can cause unpredictable changes, leading to emergence of catatonia during ECT.

Correlations between catatonic feature and temporal lobe epilepsy have been well-known. It is possible that the emergence of catatonia following ECT is mediated through the effects of ECT on temporal lobes. Thus, delivering less electrical energy in the brain and temporal lobes, in particular, can diminish the risk of catatonia emergence during ECT (14).

Also, the use of such medications as atropine in ECT procedure could contribute to the catatonia development associated with ECT (15). One may argue that catatonic features in the patient might have been related to the premedication, such as intravenous thiopental sodium and succinyl choline for ECT, and not ECT per se. Pandey and Sharma revealed that catatonia developed after ECT where no premedication was given before ECT(9).

The other probable etiology is the role of vitamin B12 deficiency in the catatonia development. Vitamin B12 deficiency has been associated with various psychiatric manifestations, such as depression, mania, and psychosis. Psychiatric symptoms can sometimes occur without hematological and neurological abnormalities and can be prodromal of vitamin B12 deficiency (16). Bram et al. reported a case of autoimmune B12 deficiency presenting as catatonia without anemia or other abnormalities where a correlation was found between the patient's B12 blood levels and catatonic symptoms over time (17).

The possible role of vitamin B12 deficiency or the extent of its role in developing catatonic state is not clear. Because the patient had been on psychotropic regimen, assessing the extent of the role of vitamin B12 deficiency was not possible. In the case of vitamin B12 deficiency in the candidates of ECT, the correction of the deficiency may prevent from the possible occurrence of catatonia in the course of ECT.

## Limitation

Possible role of ECT premedication and vitamin B12 deficiency in development of catatonia during course of ECT in this case, can make bias in the interpretation of ECT role per se.

## Conclusion

ECT is the treatment of choice for catatonia in the cases of life-threatening or refractory catatonia, development of catatonia during the course of ECT treatment is highly surprising. Our patient had some possible risk factors for developing catatonia state, such as severe vitamin B12 deficiency, concurrent use of psychotropic drugs in the course of ECT (quetiapine and biperiden), and history of long acting antipsychotic agent prior to the start of ECT. Hence, clinicians should be aware of this rare but important complication and try to reduce possible precipitating factors which make the patients susceptible to this condition.
